# Supplementary material for: Systematic review of marine environmental DNA metabarcoding studies: toward best practices for data usability and accessibility
Source: PeerJ. 2023 Mar 24;11:e14993. doi: 10.7717/peerj.14993 (PMC10042160; doi:10.7717/peerj.14993)
Supplement: Supplemental Information 1 [file peerj-11-14993-s001.pdf]

PRISMA 2020 Checklist

Section and Topic	Item #	Checklist item	Location where item is reported
**TITLE**			
Title	1	Identify the report as a systematic review.<br><br>*Systematic Review of Marine Environmental DNA Metabarcoding Studies: Toward Best Practices for Data Usability and Accessibility*	Title (1)
**ABSTRACT**			
Abstract	2	See the PRISMA 2020 for Abstracts checklist.	see below
**INTRODUCTION**			
Rationale	3	Describe the rationale for the review in the context of existing knowledge.<br><br>*"In focus, this review elevates an understudied aspect of eDNA data challenges: FAIR data principles. In scope, it centers marine studies, a specific field where conversations about data accessibility are already happening (cf. the 2020 International Virtual Conference on the use of Environmental DNA in Marine Environments and the 2022 2nd National Workshop on Marine Environmental DNA), but where no systematic reviews have tracked current data practices."*	Introduction (6)
Objectives	4	Provide an explicit statement of the objective(s) or question(s) the review addresses.<br><br>*"In the following systematic review, we analyze published marine eDNA metabarcoding studies, with a particular eye toward factors that impact the FAIRness of the underlying data, including metadata and data storage practices, in order to highlight challenges, as well as promising trends, in the continued quest toward usable and accessible eDNA data."*	Introduction (6)
**METHODS**			
Eligibility criteria	5	Specify the inclusion and exclusion criteria for the review and how studies were grouped for the syntheses.<br><br>*"Selected peer-reviewed articles met the following five criteria: 1) were published in English, 2) primarily reported novel scientific findings (no book chapters, review papers, perspective pieces, or similar), 3) collected eDNA samples directly (no modeling papers), 4) reported eDNA data from at least one water sample (no papers with samples exclusively from sediment, tissue, gut, or similar) from a marine environment (no papers with exclusively freshwater sampling), and 5) utilized a metabarcoding approach to sequence their sample(s)."*	Methods (7)
Information sources	6	Specify all databases, registers, websites, organisations, reference lists and other sources searched or consulted to identify studies. Specify the date when each source was last searched or consulted.<br><br>*"Using standard systematic review protocols (Moher et al., 2015), we conducted a literature search of peer-reviewed articles indexed in Web of Science, PubMed, and Scopus (see Figure 1). On all platforms, we used the search string ("environmental DNA" OR eDNA) AND (marine OR ocean* OR seawater OR saltwater OR sea) across titles, abstracts, and keywords to broadly identify articles using eDNA in marine environments, published up to 31 December 2020. Using this search strategy, we were only selecting for articles that self-identified as studying "environmental DNA," rather than articles using the same, or similar, methods. Because many eDNA articles are published in the journal Environmental DNA, which at the time of searching was not yet indexed in any of the databases above, we additionally searched that journal's corpus using the same search string and date range and added the returned articles to our sample."*	Methods (6-7)
Search strategy	7	Present the full search strategies for all databases, registers and websites, including any filters and limits used.<br><br>*"Using standard systematic review protocols (Moher et al., 2015), we conducted a literature search of peer-reviewed articles indexed in Web of Science, PubMed, and Scopus (see Figure 1). On all platforms, we used the search string ("environmental DNA" OR eDNA) AND (marine OR ocean* OR seawater OR saltwater OR sea) across titles, abstracts, and keywords to broadly identify articles using eDNA in marine*	Methods (6-7)

Section and Topic	Item #	Checklist item	Location where item is reported
		environments, published up to 31 December 2020. Using this search strategy, we were only selecting for articles that self-identified as studying "environmental DNA," rather than articles using the same, or similar, methods. Because many eDNA articles are published in the journal Environmental DNA, which at the time of searching was not yet indexed in any of the databases above, we additionally searched that journal's corpus using the same search string and date range and added the returned articles to our sample."	
Selection process	8	Specify the methods used to decide whether a study met the inclusion criteria of the review, including how many reviewers screened each record and each report retrieved, whether they worked independently, and if applicable, details of automation tools used in the process.<br><br>*"We utilized a two-phase screening process, first identifying potentially relevant articles from the title and abstract, and then further investigating the full text of articles passing the initial screen. During both phases, all articles were screened by two members of the research team (MS, JK, MR, or DS); any disagreements over the relevancy of a given article in either phase were resolved with the full screening team (MS, JK, MR, and DS)."*	Methods (7)
Data collection process	9	Specify the methods used to collect data from reports, including how many reviewers collected data from each report, whether they worked independently, any processes for obtaining or confirming data from study investigators, and if applicable, details of automation tools used in the process.<br><br>*"All articles were extracted by two researchers independently (of MS, JK, MR, DS); a third researcher (MS, JK, or MR) compared these extractions and resolved any differences across all elements. While the particular configuration of researchers extracting and resolving each article varied to reduce bias, one researcher (MS) either extracted or resolved every article in the sample to ensure consistency."*	Methods (9)
Data items	10a	List and define all outcomes for which data were sought. Specify whether all results that were compatible with each outcome domain in each study were sought (e.g. for all measures, time points, analyses), and if not, the methods used to decide which results to collect.<br><br>*See Methods section for details*	Methods (7-9)
	10b	List and define all other variables for which data were sought (e.g. participant and intervention characteristics, funding sources). Describe any assumptions made about any missing or unclear information.	NA
Study risk of bias assessment	11	Specify the methods used to assess risk of bias in the included studies, including details of the tool(s) used, how many reviewers assessed each study and whether they worked independently, and if applicable, details of automation tools used in the process.	NA
Effect measures	12	Specify for each outcome the effect measure(s) (e.g. risk ratio, mean difference) used in the synthesis or presentation of results.	NA
Synthesis methods	13a	Describe the processes used to decide which studies were eligible for each synthesis (e.g. tabulating the study intervention characteristics and comparing against the planned groups for each synthesis (item #5)).<br><br>*All studies were synthesized together*	NA
	13b	Describe any methods required to prepare the data for presentation or synthesis, such as handling of missing summary statistics, or data conversions.	NA
	13c	Describe any methods used to tabulate or visually display results of individual studies and syntheses.	NA
	13d	Describe any methods used to synthesize results and provide a rationale for the choice(s). If meta-analysis was performed, describe the model(s), method(s) to identify the presence and extent of statistical heterogeneity, and software package(s) used.<br><br>*"We then averaged the percent inclusion across the elements within each metadata category; these averages help show general trends across the different categories, but we do not intend to suggest that all of these metadata elements are equally important."*	Methods (8)
	13e	Describe any methods used to explore possible causes of heterogeneity among study results (e.g. subgroup analysis, meta-regression).	NA

PRISMA 2020 Checklist

Section and Topic	Item #	Checklist item	Location where item is reported
	13f	Describe any sensitivity analyses conducted to assess robustness of the synthesized results.	
Reporting bias assessment	14	Describe any methods used to assess risk of bias due to missing results in a synthesis (arising from reporting biases).	NA
Certainty assessment	15	Describe any methods used to assess certainty (or confidence) in the body of evidence for an outcome.	NA
**RESULTS**			
Study selection	16a	Describe the results of the search and selection process, from the number of records identified in the search to the number of studies included in the review, ideally using a flow diagram.<br><br>*See Figure 1*	Methods (Figure 1)
	16b	Cite studies that might appear to meet the inclusion criteria, but which were excluded, and explain why they were excluded.	NA
Study characteristics	17	Cite each included study and present its characteristics.<br><br>*See Figure 2*	Results (Figure 2)
Risk of bias in studies	18	Present assessments of risk of bias for each included study.	NA
Results of individual studies	19	For all outcomes, present, for each study: (a) summary statistics for each group (where appropriate) and (b) an effect estimate and its precision (e.g. confidence/credible interval), ideally using structured tables or plots.	NA
Results of syntheses	20a	For each synthesis, briefly summarise the characteristics and risk of bias among contributing studies.	NA
	20b	Present results of all statistical syntheses conducted. If meta-analysis was done, present for each the summary estimate and its precision (e.g. confidence/credible interval) and measures of statistical heterogeneity. If comparing groups, describe the direction of the effect.	NA
	20c	Present results of all investigations of possible causes of heterogeneity among study results.	NA
	20d	Present results of all sensitivity analyses conducted to assess the robustness of the synthesized results.	NA
Reporting biases	21	Present assessments of risk of bias due to missing results (arising from reporting biases) for each synthesis assessed.	NA
Certainty of evidence	22	Present assessments of certainty (or confidence) in the body of evidence for each outcome assessed.	NA
**DISCUSSION**			
Discussion	23a	Provide a general interpretation of the results in the context of other evidence.	Discussion (14-21)
	23b	Discuss any limitations of the evidence included in the review.	Discussion (14-21)
	23c	Discuss any limitations of the review processes used.	Discussion (14-21)
	23d	Discuss implications of the results for practice, policy, and future research.	Discussion (14-21)
**OTHER INFORMATION**			
Registration and	24a	Provide registration information for the review, including register name and registration number, or state that the review was not registered.	NA

PRISMA 2020 Checklist

Section and Topic	Item #	Checklist item	Location where item is reported
protocol	24b	Indicate where the review protocol can be accessed, or state that a protocol was not prepared.	NA
	24c	Describe and explain any amendments to information provided at registration or in the protocol.	NA
Support	25	Describe sources of financial or non-financial support for the review, and the role of the funders or sponsors in the review.	PeerJ Funding Statement
Competing interests	26	Declare any competing interests of review authors.	NA (in PeerJ Competing Interest Statement)
Availability of data, code and other materials	27	Report which of the following are publicly available and where they can be found: template data collection forms; data extracted from included studies; data used for all analyses; analytic code; any other materials used in the review.	PeerJ Data Availability Statement

Abstract-Specific Checklist

Section and Topic	Item #	Checklist item	Location where item is reported
**TITLE**			
Title	1	Identify the report as a systematic review.<br><br>*"In order to better understand these data usability challenges, we systematically reviewed 60 peer reviewed articles conducting a specific subset of eDNA research: metabarcoding studies in marine environments."*	Abstract (2)
**BACKGROUND**			
Objectives	2	Provide an explicit statement of the main objective(s) or question(s) the review addresses.<br><br>*"For each article, we characterized over 75 features across several categories: general article attributes and topics, methodological choices, types of metadata included, and availability and storage of sequence data. Analyzing these characteristics, we identified several major barriers to data accessibility, including a lack of common context and vocabulary across the articles, missing metadata, supplementary information limitations, and a concentration of both sample collection and analysis in the United States."*	Abstract (2)
**METHODS**			
Eligibility criteria	3	Specify the inclusion and exclusion criteria for the review.<br><br>*"In order to better understand these data usability challenges, we systematically reviewed 60 peer reviewed articles conducting a specific subset of eDNA research: metabarcoding studies in marine environments."*	Abstract (2); more in main text of article
Information sources	4	Specify the information sources (e.g. databases, registers) used to identify studies and the date when each was last searched.	No; in main text of article
Risk of bias	5	Specify the methods used to assess risk of bias in the included studies.	NA

PRISMA 2020 Checklist

Section and Topic	Item #	Checklist item	Location where item is reported
Synthesis of results	6	Specify the methods used to present and synthesise results.	No; in main text of article
**RESULTS**			
Included studies	7	Give the total number of included studies and participants and summarise relevant characteristics of studies.<br><br>*"In order to better understand these data usability challenges, we systematically reviewed 60 peer reviewed articles conducting a specific subset of eDNA research: metabarcoding studies in marine environments."*	Abstract (2)
Synthesis of results	8	Present results for main outcomes, preferably indicating the number of included studies and participants for each. If meta-analysis was done, report the summary estimate and confidence/credible interval. If comparing groups, indicate the direction of the effect (i.e. which group is favoured).	NA
**DISCUSSION**			
Limitations of evidence	9	Provide a brief summary of the limitations of the evidence included in the review (e.g. study risk of bias, inconsistency and imprecision).	No; in main text of article
Interpretation	10	Provide a general interpretation of the results and important implications.<br><br>*"Analyzing these characteristics, we identified several major barriers to data accessibility, including a lack of common context and vocabulary across the articles, missing metadata, supplementary information limitations, and a concentration of both sample collection and analysis in the United States. While some of these barriers require significant effort to address, we also found many instances where small choices made by authors and journals could have an outsized influence on the discoverability and reusability of data. Promisingly, articles also showed consistency and creativity in data storage choices as well as a strong trend toward open access publishing."*	Abstract (2)
**OTHER**			
Funding	11	Specify the primary source of funding for the review.	PeerJ Funding Statement
Registration	12	Provide the register name and registration number.	NA

From: Page MJ, McKenzie JE, Bossuyt PM, Boutron I, Hoffmann TC, Mulrow CD, et al. The PRISMA 2020 statement: an updated guideline for reporting systematic reviews. BMJ 2021;372:n71. doi: 10.1136/bmj.n71

For more information, visit: http://www.prisma-statement.org/
