# Supplementary material for: Systematic review of marine environmental DNA metabarcoding studies: toward best practices for data usability and accessibility
Source: PeerJ. 2023 Mar 24;11:e14993. doi: 10.7717/peerj.14993 (PMC10042160; doi:10.7717/peerj.14993)
Supplement: Supplemental Information 2 — A document detailing the rationale for conducting this systematic review and its contributions. [file peerj-11-14993-s002.docx]

**Systematic Review Rationale**

There are a growing number of initiatives concerned with data usability and accessibility of eDNA data, which could be informed by more information about how metabarcoding projects manage data challenges in practice. We conducted this systematic review to better understand factors that impact the FAIRness of the underlying data in marine eDNA metabarcoding studies, including metadata and data storage practices, in order to highlight challenges, as well as promising trends, in the continued quest toward usable and accessible eDNA data.

**Systematic Review Contribution**

While many studies have addressed eDNA data concerns in the context of reproducibility, far fewer have studied the accessibility of eDNA data (e.g. Nicholson et al., 2020 for freshwater), and none focused on marine environments. This systematic review elevates an understudied aspect of eDNA data challenges: FAIR data principles. Additionally, it centers a particular context—marine eDNA research—where conversations about data accessibility are already happening (cf. the 2020 International Virtual Conference on the use of Environmental DNA in Marine Environments and the 2022 2nd National Workshop on Marine Environmental DNA), but where no systematic reviews have tracked current data practices.
